# BμG@Sbase—a Microarray Database and Analysis Tool

**DOI:** 10.1002/cfg.197

**Published:** 2002-08

**Authors:** Adam A. Witney, Jason Hinds

**Affiliations:** Bacterial Microarray Group Department of Medical Microbiology St George's Hospital Medical School Cranmer Terrace London SW17 0RE UK

## Abstract

The manufacture and use of a whole-genome microarray is a complex process and it is essential that all data surrounding the process is stored, is accessible and can be easily associated with the data generated following hybridization and scanning. As part of a program funded by the Wellcome Trust, the Bacterial Microarray Group at St. George's Hospital Medical School (BμG@S) will generate whole-genome microarrays for 12 bacterial pathogens for use in collaboration with specialist research groups. BμG@S will collaborate with these groups at all levels, including the experimental design, methodology and analysis. In addition, we will provide informatic support in the form of a database system (BμG@Sbase). BμG@Sbase will provide access through a web interface to the microarray design data and will allow individual users to store their data in a searchable, secure manner. Tools developed by BμG@S in collaboration with specific research groups investigating analysis methodology will also be made available to those groups using the arrays and submitting data to BμG@Sbase.


**Comparative and Functional Genomics**
*Comp Funct Genom* 2002; **3**: 369–371.
Published online in Wiley InterScience (www.interscience.wiley.com). **DOI:** 10.1002/cfg.197

Conference Review

### BμG@Sbase — a microarray database and analysis tool

Adam A. Witney* and Jason Hinds
*Bacterial Microarray Group, Department of Medical Microbiology, St George's Hospital Medical School, Cranmer Terrace, London SW17 0RE, UK*

**Correspondence to:
Adam A. Witney, Bacterial Microarray Group, Department of Medical Microbiology, St George's Hospital Medical School, Cranmer Terrace, London SW17 0RE, UK.
E-mail: a.witney@sghms.ac.uk*

Received: 10 June 2002
Accepted: 12 June 2002

### Abstract

**The manufacture and use of a whole-genome microarray is a complex process and it is essential that all data surrounding the process is stored, is accessible and can be easily associated with the data generated following hybridization and scanning. As part of a program funded by the Wellcome Trust, the Bacterial Microarray Group at St. George's Hospital Medical School (BμG@S) will generate whole-genome microarrays for 12 bacterial pathogens for use in collaboration with specialist research groups. BμG@S will collaborate with these groups at all levels, including the experimental design, methodology and analysis. In addition, we will provide informatic support in the form of a database system (BμG@Sbase). BμG@Sbase will provide access through a web interface to the microarray design data and will allow individual users to store their data in a searchable, secure manner. Tools developed by BμG@S in collaboration with specific research groups investigating analysis methodology will also be made available to those groups using the arrays and submitting data to BμG@Sbase. Copyright © 2002 John Wiley & Sons, Ltd.**

**Keywords:** **microarray; bioinformatics; database**

### Introduction

The Bacterial Microarray Group at St. George's Hospital Medical School (BμG@S) has been funded by The Wellcome Trust to generate whole-genome microarrays for 12 bacterial pathogens. The arrays will be made available for collaborative research with groups around the UK and the rest of Europe. The approach taken by the BμG@S group is to generate PCR products for all genes of one strain of the specific organism plus any additional genes from other strains not found in the first. These are then verified by gel electrophoresis, purified and arrayed on coated glass slides.

A large amount of data is collected throughout the process of manufacturing the arrays, e.g. the sequences of the primers used to generate the PCR products, the conditions under which the PCR products were amplified and the band intensity of the PCR product when verified by gel electrophoresis. Such data is essential when troubleshooting the manufacturing process but it is also extremely useful during the analysis phase of a microarray experiment; it is helpful to know which portion of a gene has been amplified and printed onto the slide, and this becomes crucial when identifying gene deletions by DNA against DNA hybridization comparisons. Also, the power of the subsequent analysis of a microarray experiment can be further enhanced if the scanned experimental data can be linked to this kind of gene-specific meta-data.

### Challenges

If one considers a single microarray slide with 4000 gene-specific PCR products arrayed, which has been scanned at two wavelengths, analysis using the software package ImaGene™ [5] would generate 10 data points per spot, per wavelength (all of which may not be required currently, but may be in the future), resulting in 80 000 data points per slide. Subsequently, for an experiment using 10–20 slides we can very easily be trying

Copyright © 2002 John Wiley & Sons, Ltd.

to keep track of well over a million data points. Such a large amount of data pushes the limits of spreadsheets for the management of the data, and so reduces the ability to query the data in a useful fashion.

In addition, if one includes the information describing the biological samples used in the hybridization and the array meta-data, it is clear that the whole microarray process generates large amounts of very complex data. The collection and storage of the data in a useful fashion, such that it can be easily queried, is not straightforward. A relational database system is ideal for this purpose, as it can be built to suit the complexity that is required. Several groups have developed custom relational microarray database schemas [3], and many of these are publicly available. However, the flexibility of the technology to build relational databases means that often they are built for the needs of a particular user environment. The BμG@S group is therefore developing a system (BμG@Sbase) to provide informatic support to collaborative research group users.

### Microarray standards

The complex nature of the microarray process gives rise to a vast array of terminology, such that to allow for effective communication of experimental data it is important to develop a standard language. Advances in the development of standards in the field of microarray applications are being led by the Microarray and Gene Expression Database group (MGED [6]); an international collaboration of academic and industrial scientists started in 1999. The MGED group set up four working groups to approach different aspects of microarray informatics. The minimal information about a microarray experiment (MIAME) group aims to establish the minimum information required to be reported, such that an independent researcher can understand and repeat the experiment. Version 1.0 of the MIAME document has been published [2], is continually being developed and is constantly maturing. The MAGE group is focusing on the development of the Microarray and Gene Expression Object Model and Markup Language (MAGE-OM and MAGE-ML), a standard format for exchange of microarray data, and the development of a software toolkit (MAGEstk) to enable the writing of code (currently in Perl and Java) to read and write MAGE-ML. Separate groups have also been tasked to develop a microarray specific ontology and to recommend standard normalization methods and reporting of such methods. The standards discussed are constantly evolving and are beginning to be used in real-world applications; the European Bioinformatics Institute (EBI) is developing ArrayExpress [1], a public repository based on the MIAME standard, and the National Center for Biotechnology Information (NCBI) is incorporating MIAME into their public microarray repository, Gene Expression Omnibus (GEO) [4]. The array design data currently stored within BμG@Sbase is compatible with the MAGE-OM and we hope to be able to capture experimental data according to the developed standards.

### BμG@Sbase

The purpose of BμG@Sbase is to provide as much information about the arrays as is useful. Also, as discussed above, the storage and manipulation of hybridization and post-hybridization data is not straightforward and so, rather than all users setting up their own local database systems, we will enable collaborative research groups using the BμG@S arrays to store their data in BμG@Sbase in a secure user area. In addition, users will have access to tools built by the BμG@S group to further interrogate the array and experimental data. The system allows users to interrogate the array information in different ways, e.g. one can easily BLAST a query sequence against all the sequences that are printed onto a particular array; this can be useful when looking for cross-hybridization between spots or to see if a previously unknown gene is represented on the array. Also, the user is able to find specific gene description information for any particular spot on a specific array.

This approach has many advantages for both the collaborative users and BμG@S; the users do not have to purchase, set up and administer a local database server and software (which is not a trivial task), the users have access to up-to-date information about the arrays without having to perform downloads from a central BμG@S site, and similarly BμG@S will be able to easily disseminate up-to-date information to multiple users. Users will be able to store their data in a secure, robust and

Copyright © 2002 John Wiley & Sons, Ltd. 

searchable manner, and have access to analysis tools that will be built and added onto BμG@Sbase by the BμG@S group through their ongoing collaborations with research groups developing microarray analysis methodology. Users will also be able to compare analysed data with that of other collaborating groups, and to easily export their experimental data and the array and experimental meta-data in MAGE-ML format for transport into other database systems and software applications, and (upon publication) for submission to public microarray repositories. In addition, BμG@S will be able to keep track of when and where arrays have been distributed and so ensure good use of its resources.

### The future

Microarray data analysis is a constantly evolving field and in time more powerful analysis methodology and tools will be developed that will be able to ask different questions of the data. Therefore, it is very important that the data being generated now will be accessible for the more advanced tools of the future. BμG@S will develop analysis and visualization tools and incorporate analysis methods, as they are introduced by collaborators and other researchers in the field.

### Acknowledgements

The authors wish to thank all the members of the BμG@S group and the Wellcome Trust for funding the BμG@S facility. BμG@Sbase is derived from a microarray database schema developed by AAW and colleagues at the Naval Medical Research Center, MD, USA, with funding from USAMRMC and the US Department of the Navy. Improvements to database schema and interactivity are being developed under Cooperative Research and Development Agreement with the Naval Medical Research Center.

### References

1. Arrayexpress public microarray data repository: **http://www.ebi.ac.uk/microarray/ArrayExpress/arrayexpress.html**
2. Brazma A, Hingamp P, Quackenbush J, *et al*. 2001. Minimum information about a microarray experiment (MIAME) — toward standards for microarray data. *Nature Genet* **29**(4): 365–371.
3. Gardiner-Garden M, Littlejohn TG. 2001. A comparison of microarray databases. *Brief Bioinform* **2**(2): 143–158.
4. Gene Expression Omnibus: **http://www.ncbi.nlm.nih.gov/geo/**
5. ImaGene software package: **http://www.biodiscovery.com**
6. Microarray and Gene Expression Database group: **http://www.mged.org**

Copyright © 2002 John Wiley & Sons, Ltd. 
